# Time-Restricted Eating as a Nutrition Strategy for Individuals with Type 2 Diabetes: A Feasibility Study

**DOI:** 10.3390/nu12113228

**Published:** 2020-10-22

**Authors:** Evelyn B. Parr, Brooke L. Devlin, Karen H. C. Lim, Laura N. Z. Moresi, Claudia Geils, Leah Brennan, John A. Hawley

**Affiliations:** 1 Exercise and Nutrition Research Program, Mary MacKillop Institute for Health Research, Australian Catholic University, 215 Spring Street, Victoria 3000, Australia; karen.lim@acu.edu.au (K.H.C.L.); john.hawley@acu.edu.au (J.A.H.); 2 Department of Dietetics, Nutrition and Sport, La Trobe University, Plenty Road and Kingsbury Drive, Bundoora, Victoria 3086, Australia; b.devlin@latrobe.edu.au; 3 School of Behavioural and Health Sciences, Australian Catholic University, Locked Bag 4115, Fitzroy, Victoria Melbourne 3065, Australia; lauranichole1204@hotmail.com (L.N.Z.M.); Claudia.geils@myacu.edu.au (C.G.); leah.brennan@latrobe.edu.au (L.B.); 4 School of Psychology and Public Health, La Trobe University Albury-Wodonga Campus, 133 McKoy Street, West Wodonga, Victoria 3690, Australia

**Keywords:** intermittent fasting, dietary adherence, energy restriction, psychological well-being, cognitive function, glycaemic control

## Abstract

Individuals with type 2 diabetes (T2D) require a long-term dietary strategy for blood glucose management and may benefit from time-restricted eating (TRE, where the duration between the first and last energy intake is restricted to 8–10 h/day). We aimed to determine the feasibility of TRE for individuals with T2D. Participants with T2D (HbA1c >6.5 to <9%, eating window >12 h/day) were recruited to a pre-post, non-randomised intervention consisting of a 2-week Habitual period to establish baseline dietary intake, followed by a 4-weeks TRE intervention during which they were instructed to limit all eating occasions to between 10:00 and 19:00 h on as many days of each week as possible. Recruitment, retention, acceptability, and safety were recorded throughout the study as indicators of feasibility. Dietary intake, glycaemic control, psychological well-being, acceptability, cognitive outcomes, and physiological measures were explored as secondary outcomes. From 594 interested persons, and 27 eligible individuals, 24 participants enrolled and 19 participants (mean ± SD; age: 50 ± 9 years, BMI: 34 ± 5 kg/m^2^, HbA1c: 7.6 ± 1.1%) completed the 6-week study. Overall daily dietary intake did not change between Habitual (~8400 kJ/d; 35% carbohydrate, 20% protein, 41% fat, 1% alcohol) and TRE periods (~8500 kJ/d; 35% carbohydrate, 19% protein, 42% fat, 1% alcohol). Compliance to the 9 h TRE period was 72 ± 24% of 28 days (i.e., ~5 days/week), with varied adherence (range: 4–100%). Comparisons of adherent vs. non-adherent TRE days showed that adherence to the 9-h TRE window reduced daily energy intake through lower absolute carbohydrate and alcohol intakes. Overall, TRE did not significantly improve measures of glycaemic control (HbA1c −0.2 ± 0.4%; *p* = 0.053) or reduce body mass. TRE did not impair or improve psychological well-being, with variable effects on cognitive function. Participants described hunger, daily stressors, and emotions as the main barriers to adherence. We demonstrate that 4-weeks of TRE is feasible and achievable for these individuals with T2D to adhere to for at least 5 days/week. The degree of adherence to TRE strongly influenced daily energy intake. Future trials may benefit from supporting participants to incorporate TRE in regular daily life and to overcome barriers to adherence.

## 1. Introduction

Time-restricted eating (TRE) is a dietary strategy in which food intake is reduced to a "window" of 8–12 h/day during waking hours. TRE has been demonstrated to be a simple and practical behavioural intervention that can reduce energy intake in those with obesity or metabolic syndrome [1,2,3,4,5], although TRE may be more difficult in older (>65 years) populations [6]. Controlled studies of isoenergetic TRE have shown beneficial effects on glucose tolerance, insulin sensitivity, and 24-h glucose profiles in individuals at risk of developing type 2 diabetes mellitus (T2D) [7,8,9] as well as reductions in appetite in persons with overweight/obesity [10]. The proposed mechanism for improved metabolic health with TRE, despite no restriction in energy intake, is temporal alignment between energy consumption and normal diurnal rhythms of hormones involved in metabolic processes and energy regulation [11,12,13]. For individuals with T2D, diurnal regulation of glucose is altered such that highest peaks in glucose occur in the morning [14], compared to in the afternoon/evening in those with normoglycemia [15]. Further, skipping breakfast increases glycaemic responses to lunch and dinner in individuals with T2D [16]. Thus, the most effective TRE approach for individuals with T2D may be delaying breakfast to mid-morning. Additionally, as late night eating is a common, modifiable factor of the modern lifestyle [17], which adversely affects glycaemic control [18,19], strategies such as TRE which reduces late night eating [3] may improve glycaemic control in individuals with T2D. Specifically, data from a 2-week pilot study of intermittent fasting (equivalent to 4–6 h TRE) indicated that individuals with T2D were able to restrict their energy intake to ~8 h per day for two weeks and was beneficial for glycaemic management, but 40% indicated they would not continue with the dietary restriction [20]. However, no study to date has comprehensively investigated the feasibility of 9-h TRE in individuals with T2D, and the effects on dietary intake and glycaemic control.

T2D is a chronic multifactorial disorder with complications closely linked to the duration since onset and the success of glycaemic control [21]. In clinical research trials, individuals with T2D improve both physiological and psychological outcomes when implementing dietary interventions [22,23]. However, long-term adherence to such dietary interventions is poor, with individuals with T2D reporting that maintenance of dietary change is more burdensome than medical therapy [24]. Due to the complex nature of the pathophysiology, cause and treatment of T2D, and the importance of effective self-management, T2D is considered one of the most psychologically demanding medical conditions in adults [25]. Psychological well-being is important not only for individual welfare, but also due to its association with self-management and dietary adherence [26]. To determine long-term sustainability and safety of TRE, it is critical to establish the impact of TRE on psychological factors as well as dietary adherence, glycaemic control, and physiological outcomes.

Previous investigations have explored the mechanisms by which aligning the timing of eating with 24-h diurnal rhythms can improve metabolic health [7,8,13]. Recently, several studies have evaluated the feasibility of TRE in real-world settings [1,5,27] but with limited exploration as to how TRE affects dietary intake (i.e., energy and micronutrient composition) and physiological variables (i.e., body composition and blood pressure). It is important to understand individuals experience of implementing TRE, including feedback regarding the ability to adhere to the TRE window, general acceptability of this eating approach, and the potential psychological effects of focusing on the timing of eating. Accordingly, the aims of this study were firstly, to determine the feasibility (recruitment, compliance, attitudes, and acceptability) of adhering to TRE for individuals with diet- or medication-controlled T2D; and secondly, to explore the effects of TRE on dietary changes, glycaemic control, physiological outcomes, and psychological wellbeing. We hypothesized that TRE would be feasible and acceptable to those with T2D and have no adverse outcomes or reactions.

## 2. Materials and Methods 

### 2.1. Study Design

This study was a feasibility study conducted at Australian Catholic University’s Melbourne campus between July 2018 and November 2019. Ethical approval was obtained from the ACU Human Research Ethics Committee (2018-75H) and was prospectively registered on the Australia New Zealand Clinical Trial Registry (ACTRN12618000938202). The study was conducted in accordance with the Declaration of Helsinki and all participants provided written informed consent prior to participation.

The study was a pre-post non-randomized design whereby participants completed a 2-week “Habitual” baseline period of dietary recordings followed by a 4-week TRE intervention period where they were instructed to limit all eating occasions to between 10:00 and 19:00 h on as many days each week as possible. No dietary advice around food type, quality, or portion sizes was provided. Participants were requested to take photos at every eating occasion and complete daily food diaries throughout both the Habitual and TRE intervention periods. The primary outcomes for the study are reported here, including the feasibility of recruitment (number of persons screened compared to final enrolments); retention rate (number of participants enrolled compared to the number of participants who finish the final study measures); safety (participant report of adverse events, i.e., self-report of any gastrointestinal, nausea, dizziness, or other events as a result of the dietary intervention); compliance to the dietary intervention assessed using a combination of participant self-report and photo images of meal times (with time stamps); and acceptability (attitudes/opinions/barriers assessed in a qualitative interview at the completion of the intervention (i.e., after 4 weeks of TRE)). We also explored glycaemic control (via mixed meal tolerance test (MMTT), fasting blood profiles (e.g., glycated haemoglobin (HbA1c), glucose, insulin and lipids), physiological (e.g., body mass, composition, blood pressure (BP), and resting metabolic rate (RMR)) and psychological outcomes (e.g., quality of life, depression, anxiety and stress, eating behaviours, sleep quality and cognitive function, assessed using validated self-report questionnaires) pre and post the TRE intervention period.

### 2.2. Participants

Individuals with T2D (aged 35–65 years, body mass index (BMI) between 25 and 45 kg/m^2^) were recruited using targeted campaigns of flyers/posters, social media advertisements, databases from previous studies and via a research participant recruitment company (Trialfacts, Melbourne, Australia). Initial eligibility screening was conducted by telephone and comprised of medical history, age, and estimates of height and weight (to calculate BMI). Respondents were eligible if they met the following criteria: diagnosed (by a GP/endocrinologist) with T2D, with an HbA1c between 6.5 and 9%, and either diet-controlled or taking a maximum of two oral hypoglycaemic agents (excluding sulphonylureas, insulin, and GLP-1 agonists); and currently consuming food (i.e., dietary intake) over a period of 12 h or more, habitually (i.e., self-reported on 5 of 7 days/week). 

Potential participants were excluded based upon the following criteria: not a regular (≥5 days/week) breakfast consumer; could not operate the camera function on a smart phone; taking glucose lowering medications which are contraindicatory for fasting [28] (i.e., sulphonylureas, or insulin) or requiring injecting (i.e., GLP-1 agonists); reported to be following a strict diet (i.e., vegan, coeliac/gluten free, or ketogenic); had participated in regular fasting (defined as fasting ≤16 h/day or having completed twelve 24-h fasts within the past year); participating in shift work (i.e., >3 h between 22:00 and 05:00 h for 1 day/week (>50 days per year)); not weight stable (>5 kg change over last 3 months); on prescribed medications required to be taken with food in the early morning or late evening or taking other prescribed medications for <3 months; current smoker (tobacco, nicotine, or marijuana) or within 3 months of quitting; women who were pregnant, breastfeeding (within 24 weeks); history of psychotic disorder, current diagnosis of other major psychiatric illness (e.g., mood disorder, eating disorder, substance use disorder; does not include depression); psychopharmacological treatment that has not been stable for more than 3 months; taking medications known to promote weight gain, weight loss or interact with glucose metabolism (i.e., corticosteroids); diagnosed gastrointestinal conditions, surgery (i.e., bariatric) or impaired nutrient absorption; or antibiotic use in previous 3 months. 

Potentially-eligible participants were invited to meet with the principal researcher to provide informed consent and complete questionnaires including morning-eveningness questionnaire self-assessment (MEQ-SA) [29,30] and Eating Attitudes Test-26 EAT-26; [31] to identify circadian preferences and tendencies for eating disorders, respectively. The two participants demonstrating an elevated EAT-26 score (i.e., ≥20) met with a dietitian to clarify these scores. In both cases they were attributed to diabetes-related dietary stress rather than eating disorders, so the participants remained in the study.

### 2.3. Measurement Visits

**Figure 1:**
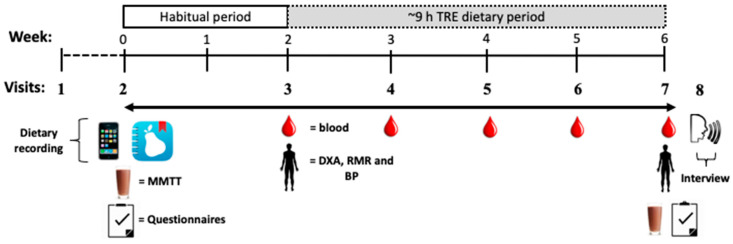
Schematic overview of study protocol. Participants completed a two-week Habitual baseline monitoring period (Week 0–2), immediately followed by a four-week intervention period of time-restricted eating (TRE; Week 2–6; consuming energy within 10:00 and 19:00 h on as many days of the week as possible) with weekly visits to the research team. Dietary recordings were collected throughout the entire 6-week period. A mixed-meal tolerance test (MMTT) and psychological questionnaires were conducted at the beginning of the Habitual (Visit 2) and end of the TRE (Visit 7) periods. Weekly, fasted blood samples were obtained from Visits 3–7. Physiological measures (body composition (dual-energy x-ray absorptiometry (DXA)), resting metabolic rate (RMR) and blood pressure (BP)) were conducted at the end of the Habitual (Visit 3) and TRE (Visit 7) periods, with a qualitative interview at the end of the TRE period (Visit 8).

Following informed consent and enrolment, participants attended the laboratory between 07:00 and 09:00 h after a >10 h fast for a baseline MMTT. A fasting blood sample (4 mL, EDTA) was collected from an indwelling cannula inserted into an antecubital vein prior to consumption of the MMTT drink (50% CHO, 30% fat, 20% protein; Sustagen Hospital Chocolate with Fibre with full fat (3.5%) milk) which was 20% of estimated total daily energy requirements, calculated using the Schofield equation (activity factor 1.3) [32]. Following the MMTT drink consumption, blood samples (4 mL, EDTA) were obtained every 30 min for 2 h. During the MTTT, psychological and cognitive testing was performed between blood sampling points. At the end of the MMTT, participants were given a handbook for dietary recordings and instructions for taking photos of all meals and snacks and were asked to complete a 2-week “Habitual” period of baseline monitoring. After the 2-week, at Visit 3 (Figure 1), participants reported to the laboratory after a 10-h fast for measures of body composition (dual-energy x-ray absorptiometry (DXA); GE Lunar iDXA Pro, enCORE software Version 16) and resting energy expenditure (REE; TrueOneRMR, Parvo Medic, Sandy, UT, USA), as previously reported [33], and seated blood pressure (BP; ProBP 3400, Welch Allan Inc, Skaneateles Falls, NY, USA), prior to a fasting blood sample via venepuncture. Dietary entries where checked for completeness with questions asked to clarify any information from written records, EDD, or photos. Participants then reported to the laboratory once per week for 4-weeks for ~30 min for a fasting blood sample, to check dietary entries and were queried with regards to any medical issues that may have occurred (Visits 4–6). The fasted physiological measurements (DXA, RMR, and BP), MMTT, psychological, and cognitive testing were repeated at the end of the 4-weeks of TRE (Visit 7). Within 0–4 days of the end of the fourth week (Visit 8), a qualitative interview was conducted by a researcher (EBP) to understand attitudes, barriers and adherence, with written notations and voice recordings (VoiceRecorder app for iOS, Tapmedia Ltd., London, UK). At the conclusion of the study, participants were gifted a $250 supermarket voucher to thank them for their participation.

### 2.4. Dietary Recordings and Analysis

For the entire study period (6 weeks), participants were asked to record all food and drink consumed using their choice of paper-based handbook or using the publicly available phone app EasyDietDiary (EDD; Xyris Software, Brisbane, Australia). The paper- or app-recorded intakes were entered into FoodWorks 9 software (Xyris, Brisbane, Australia) using the AUSNUT 2011-13 nutrient database (https://www.foodstandards.gov.au/science/monitoringnutrients/ausnut/pages/default.aspx) and all entries were reviewed/screened by one researcher (KHCL). In conjunction with recording their diet, participants were asked to photograph each eating/drinking occasion using their own mobile device. The photos were then stored in a shared folder with the primary investigator or were transferred at each measurement visit. On occasions where participants missed taking a photo, they were encouraged to manually record the time of food or drink consumption in the paper-based handbook or in the notes section of the EDD app (as the app does not allow for time data or time-stamped photos). Photos were encouraged over written records as time-stamp data generated by mobile devices were more accurate than the estimated written times (e.g., writing 13:00 h vs. time-stamp 13:11 h). Time-stamp data were extracted from raw photo files using Directory Lister Pro v2.36 (KRKsoft.com, Krakow, Poland) and researchers viewed and recorded the contents of each photo to match time-stamps with the corresponding meals/snacks recorded in the EDD app or paper diary. These time-stamps and participant-written times were also entered into FoodWorks 9. At face-to-face appointments, where necessary, participants were asked by one of the research team to clarify any ambiguous food entries or food photos for clarity and accuracy of dietary recordings. Regardless of method of recording dietary entry, participants were asked to self-report whether they were adherent on each day of the TRE intervention by circling Yes or No to the question, “*Were you able to adhere to the 10 am to 7 pm time-restriction for eating and caffeine intake today?*”.

Food and drink intake were grouped into eating occasions using the definition that best predicted variance in energy intake [34], which is food/drink consumed within a 15-min window with energy content ≥210 kJ. In the paper diaries, participants were asked to report foods eaten during “Early morning”, “Breakfast”, “Morning tea”, “Lunch”, “Afternoon tea”, “Dinner” or “Supper”. The EDD app asked participants to report foods as “Breakfast”, “Lunch”, “Dinner” or “Snacks & Drinks”. With the exception of “Snacks & Drinks”, median time of these meals/snacks by weekend/weekday and Habitual/TRE was calculated. For example, median times for breakfast on a Habitual weekday, Habitual weekend, TRE weekday, and TRE weekend were calculated for each participant. Where participants had recorded the composition of a type of meal/snack but timing was not available, the corresponding median time was applied (e.g., a participant’s median time of habitual weekday breakfast was used if the actual time of such a breakfast was unavailable). Where time was available but not meal type (i.e., a photo was available but food was categorised as “Snacks & Drinks’’) and the 15-min and ≥210 kJ criteria were not met, the food was classified based on the adjacent meals/snacks (e.g., a food entry with a photo taken at 15:20 h would be classified as ‘Afternoon tea’ if it fell between the participant’s lunch and dinner that day). Further, to allow for photos taken just prior or following consumption, a 15-min buffer was applied, so that time-stamps between 09:45 and 09:59 h and 19:01 and 19:15 h were considered as falling within the TRE time window. To account for days where food consumption continued past midnight, one ‘day’ of dietary data was defined as starting from the first eating occasion until the last eating occasion of the day (e.g., “Supper” at 01:30 h on a Tuesday was grouped with Monday’s intake). In this group of 19 participants, the earliest first eating occasions of the day occurred at 05:15 h and the latest occurred at 03:36 h. 

### 2.5. Biochemical Analysis

Upon collection, blood samples (EDTA) were inverted and blood lipids (triglycerides, total, HDL, and LDL cholesterol) were analyzed in whole blood at the time of collection using a Cobas b 101 instrument (Roche Diagnostics Ltd., Basel, Switzerland). At Visits 3 and 7, glycated haemoglobin (HbA1c) was also measured from whole blood using the Cobas b 101 instrument prior to centrifugation. The remaining samples were spun at 1800× *g* for 10 min at 4 °C to obtain plasma samples which were then frozen at −80 °C for later analysis. Glucose concentrations were measured in duplicate from thawed plasma samples using YSI 2900 analyzer (YSI Life Sciences, Yellow Springs, OH, USA; coefficient of variation (CV) < 1.0%). Insulin concentrations were measured using thawed plasma with a commercially available ELISA (Alpco Ltd., Windham, NH, USA), in duplicate with an intra-assay CV of 3.8%. Total area under the curve (AUC) was calculated for venous glucose and insulin concentrations using the trapezoid method using GraphPad Prism (Version 7.01, GraphPad Software Inc., San Diego, CA, USA).

### 2.6. Psychological Questionnaires and Analysis

Psychological Wellbeing: The Depressive Anxiety Stress Scale 21 (DASS) was used to measure participants negative emotional states of depression, anxiety, and stress [35]. The AQoL-8D was used to measure participants health-related quality of life [36]. The Pittsburgh Sleep Quality Index (PSQI) was used to measure participants sleep quality and disturbances [37]. The Eating Disorders Examination Questionnaire (EDE-Q) was used to measure participants behaviour and attitude towards eating [38]. The Clinical Impairment Assessment questionnaire (CIA) was used to measure participants psychological impairment resulting from eating disorder symptoms [39]. All psychological measures assessed variables over the previous 7 (i.e., DASS, AQoL-8D) to 28 days (CIA, EDE-Q, PSQ) and have been validated and used in both community and clinically-indicated samples.

Cognitive Functioning: The Cogstate Brief Battery (CBB) was used to measure participants cognitive functioning across four domains including processing speed (detection task—measuring speed of performance), attention (identification task—measuring speed of performance), visual learning (one card learning task—measuring accuracy of performance) working memory (two back task—measuring accuracy of performance) [40], and executive functioning (Groton Maze Learning Task—measuring errors in performance). Lower scores on the Groton Maze Learning, identification and detection tasks indicate improved cognitive performance. Higher scores on the one card learning and two back tasks indicate improved cognitive performance. These constructs were chosen as they have been shown to be impacted by metabolic disorders, and/or lifestyle interventions [41]. These measures were selected as they have demonstrated validity, reliability, stability and sensitivity in healthy and disordered populations. Each task has been developed to be brief, simple and able to be repeated without giving rise to practice effects [42].

All psychological data was checked for accuracy and completeness through routine data cleaning and management procedures. Intervention efficacy was assessed through comparison of the outcomes pre- and post-intervention using a series of repeated measures analyses. AQoL-8D results were calculated as weighted psychometric scores to allow comparison before and after intervention [43]. Cognitive data was transformed according to Cogstate data handling procedures prior to analysis [42].

### 2.7. Qualitative Interviews and Analysis

Semi-structured interviews consisting of 13 open-ended questions were conducted by one researcher (EBP) to gain insight and understanding of the attitudes of participants after the 4-weeks TRE intervention period. The questions within the interviews were developed based on a previous intervention ([44]; see Supplementary Material: Qualitative Interview Questions). Written and recorded responses were collated and analysed by one researcher (CG). The audio-recorded interviews were imported verbatim into NVivo (NVivo qualitative data analysis software, Version 10, QSR International 14 Pty Ltd., VIC, Australia) and supplemented with the interviewer’s written notes when required. Data were then explored using thematic analysis [45]. Data from the interviews were first read and key preliminary findings were highlighted and noted. Participants’ responses were then coded in an inductive, exploratory manner. These codes were then grouped into common themes and reviewed and adjusted where necessary. Post-analysis verification of themes was conducted between three investigators (CG, LB, and EBP). Quotations from original responses provide support for the findings.

### 2.8. Statistical Analyses

As this was a feasibility study, it was not possible to conduct power analyses to determine sample size requirements. However, due to the varied tolerability to an intermittent fasting intervention (equivalent to 4–6 h TRE) in individuals with T2D reported in a previous 2-week intervention with an *n* = 10 sample size [20], we set out to recruit 24 individuals, with an expected dropout rate of ~15%, to complete 20 individuals. All data was analysed using the IBM Statistical Package for Social Sciences (SPSS) version 25. Significance was set at *p* < 0.05 and all data are presented as mean ± SD. One-way ANOVA tests were used to compare differences between intervention (Habitual vs. TRE) periods, with mean differences and 95% confidence intervals (CI) reported for significant differences and with effect sizes (Cohen’s *d*) where appropriate. Where more than one factor was investigated (i.e., intervention period and time or proportion of adherence), linear mixed models were used with post hoc tests using Bonferroni corrections, where appropriate. For analyses of nutrition and physiological data, visual inspection of outcome variable residual plots was conducted to assess their distributions.

## 3. Results

### 3.1. Participant Recruitment and Characteristics

**Figure 2:**
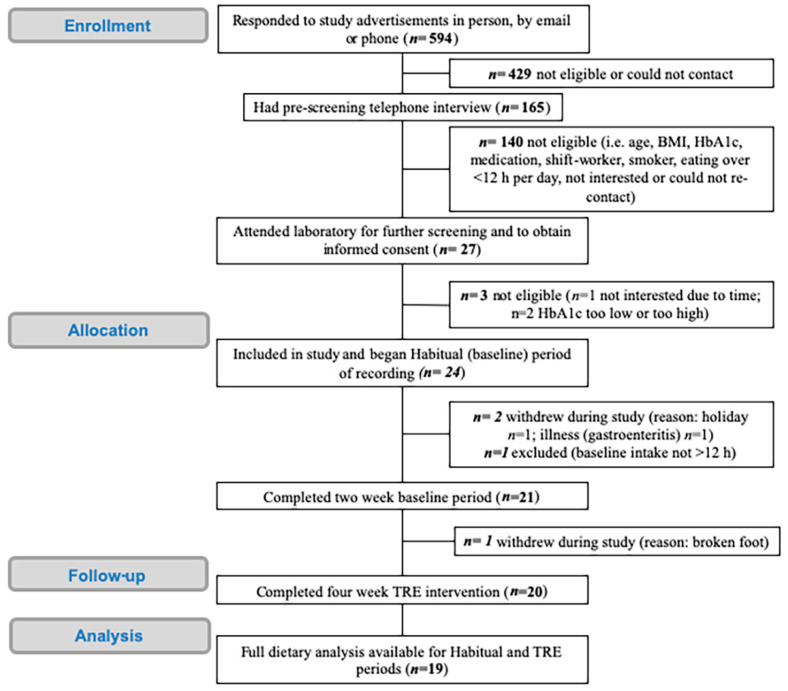
Consolidated Standards of Reporting Trials (CONSORT) flow diagram of participant inclusions.

Through multiple forms of advertising, 594 participants either contacted the research team directly (via phone or email; 25%) or answered the online screening questionnaire promoted by Trialfacts (75%) (see Figure 2). Trialfacts referred 449 respondents, of whom 417 were not eligible (93%) to participate largely due to time between first and last meal (eating window) of <12 h (40%), BMI outside of 25–45 kg/m^2^ (26%), not a regular breakfast consumer (24%), or HbA1c not between 6.5 and 9% (21%). The phone screening of the pre-screened Trialfacts referrals led to 15 of 32 participants being eligible (where 3 were uncontactable, 7 were not interested, and 7 were ineligible) and 13 (87%) were then enrolled into the study (with 2 with HbA1c too high (≥9.0%)). Of those who directly contacted the research team via phone or email (145), 133 were phone screened for further eligibility, with 121 not eligible (91%; due to medications (37%), could not recontact (16%), HbA1c not in range (15%), not interested (13%), eating window (10%) or BMI out of range (9%)), and therefore 12 participants attended the laboratory for pre-screening with 11 (92%) enrolling into the study. Thus, 24 participants were enrolled in the study and began the Habitual baseline period, which was 4% of those who expressed initial interested in the study.

Although 20 participants completed the 4-week TRE intervention period, there was insufficient dietary data (7/14 days in Habitual, 50%; and 13/28 days in TRE, 46%) for one participant. Therefore, all analysis is for the final participant cohort of 19 and participant characteristics of the cohort is presented in Table 1. Of the 19 participants, nine were not taking any hypoglycaemic medications, ten were taking biguanides (i.e., metformin), three taking SGLT-2 inhibitors and two taking DPP-4 inhibitors, with three participants taking two hypoglycaemic medications. No incidences of hypoglycaemia were reported by participants nor were any adverse events recorded during the duration of the study.

### 3.2. Dietary Analysis and Adherence

Of the 19 participants, six completed written recordings with the remainder using the electronic EDD app. Dietary data for each day were considered valid if participants recorded two or more meals/snacks. Days with a single meal/snack were valid if these records were verbally confirmed at the weekly visits. Table 2 outlines the average dietary intake throughout the Habitual and TRE periods for all participants, with individual data presented as box plots for each period in Supplementary Materials Figure S1. There were no differences in daily energy intake, macronutrient contribution to daily energy intake (relative, %), or absolute macronutrient or micronutrient intakes between Habitual and TRE period (*p* > 0.05). 

**Figure 3:**
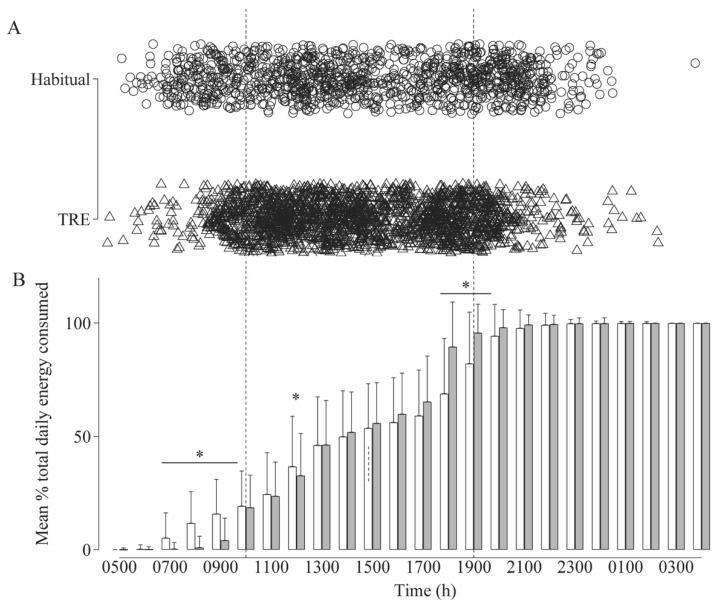
(**A**) Time of eating occasions during Habitual (○ *n* = 1089; 2 weeks) and time-restricted eating (TRE; △ *n* = 2051; 4 weeks) periods and (**B**) mean ± SD energy accumulation across the day during Habitual (unfilled bars) and TRE (filled bars). Significance (*p* < 0.05) * between periods (Habitual vs. TRE) within time points, from linear mixed model analysis with Bonferroni post hoc tests.

Participants reported a total of 3427 energy-containing meals/snacks over the 6 week recording period, of which ~92% met the ≥210 kJ energy content threshold (1089/1234 in Habitual period and 2051/2233 in TRE period, Figure 3A) to be defined as an eating occasion (EO; [34]). Participants complied with providing photos or self-reported timing for 90 ± 12% of meals/snacks (range: 63–100%; 87 ± 20% of Habitual period days and 91 ± 12% of TRE period days). When participants did not provide photos or timing of EO’s, calculations of total energy intake were reduced in the TRE period (with time data: 9013 ± 2687 kJ/d; without time data: 7590 ± 3057 kJ/d, *p* < 0.001) but was not altered in the Habitual period (with time data: 8757 ± 2764 kJ/d; without time data: 8245 ± 2682 kJ/d, *p* = 0.07).

Participants reported an average of 3.7 ± 1.2 EO’s per day during the 6 weeks. On average, TRE reduced the time window of energy consumption by 22% from 10 h 42 min to 8 h 20 min (Figure 3A). Using a previous definition of the energy consumption window derived from 95% of the times a participant consumed a meal/snack containing >5 kcal (21 kJ) [3,4], the median timespan of eating was 14 h 19 min and reduced to 11 h 30 min in the TRE period (−2 h 53 min; 95%CI: 1 h 52 min–3 h 45 min; *p* < 0.001). Mean time of first EO shifted from 09:19 h in the Habitual period to 10:32 h in the TRE period and from 20:03 h to 18:43 h for the last EO for Habitual and TRE periods, respectively. Despite the reduction in duration over which energy was consumed, the number of EOs was similar between Habitual (3.9 ± 1.3 EOs) and TRE (3.7 ± 1.1 EOs) periods. The distribution of EOs, and thus the accumulation of total energy intake, differed between the Habitual and TRE periods (Figure 3B; main interaction effect, *p* < 0.001). Delaying the start time of eating reduced the accumulated energy intake in the TRE period between 07:00 and 09:00 h and at 12:00 h (*p* < 0.05). The earlier finishing eating time corresponded with a greater proportion of energy consumption occurred between 18:00 and 19:00 h in the TRE period (*p* < 0.05). 

Prior to the prescribed TRE period, participants did not habitually restrict energy consumption to the 10:00–19:00 h window, with 0.9 ± 1.2 days meeting the TRE intake window. Adherence to the 10:00–19:00 h per day TRE window (±15 min either side) during the TRE period, as assessed by photo metadata or self-reported time of eating, was 20 ± 7 of the 28 days (range: 1–28 days; 72 ± 24%, range: 4–100% compliance), equivalent to ~5 days/week. During TRE, a greater proportion of weekdays (i.e., Monday–Friday) were deemed adherent to the prescribed TRE period (77% (288/376 weekdays)) compared to Saturdays and Sundays (61% (90/148 weekend days)). A greater proportion of daily non-adherence was due to food/drink being consumed after 19:00 h (60%; 87/146 non-adherent days), while 23% (33/146 days) was due to food/drink being consumed before 10:00 h and 17% due to non-adherent in the morning and evening (before 10:00 h and after 19:00 h).

Participants failed to report whether they were adherent or not in their written handbook on 14% of occasions (Table 3). On the 453 instances where adherence was self-reported (Yes/No), 17% of self-report was inaccurate compared to time-stamped adherence (09:45–19:15 h). Participants reported non-adherence on 23/325 occasions (8%) when the time-stamped dietary data indicated they were adherent. In contrast, a larger proportion of participants reported they were adherent on 55/128 occasions (43%) when the time-stamped dietary data indicated they did not adhere. 

Considering the variability in self-report responses, adherence data for dietary analysis has been calculated using the time-stamped dietary data. When comparing the days where participants did adhere to the time-restriction of 09:45–19:15 h, total energy intake was significantly lower (−1033 kJ, 95%CI: −1544 to −523 kJ, *p* < 0.001; Table 4). That is, when participants were adherent to the prescribed TRE period they consumed lower absolute intakes of carbohydrate (−49 g/d, 95%CI: −64 to −33 g/d), sugar (−10 g/d, 95%CI: −17 to −4 g/d), fibre (−3 g/d, 95%CI: −5 to −1 g/d), alcohol (−7 g/d, 95%CI: −10 to −5 g/d) and potassium (−201 mg/d, 95%CI: −389 to −14 mg/d), which coincided with higher relative intakes of protein (+2%, 95%CI: 1–3%) and fat (+4%, 95%CI: 2–7%) and lower relative intakes of carbohydrate (−5%, 95%CI: −7 to −2%) and alcohol (−2%, 95%CI: −3 to −1%). To assess how adherence to the 10:00–19:00 h TRE period affected dietary intake over the 4-w in comparison to habitual intakes, the proportion of time spent adherent in the TRE period (from time-stamped data; median adherence 77% (interquartile range: 54–93%) was modelled against nutrient intakes in the habitual vs. TRE periods. Only carbohydrate intake was associated with TRE adherence, with lower intakes reported by the more adherent participants. Specifically, during the TRE phase, participants in the lower quartile of adherence (54% adherence) consumed 204 g/d (95%CI: 175–232 g/d) carbohydrate compared to 151 g/d (95%CI: 120–181 g/d) carbohydrate in the upper quartile (93% adherence), a difference of 53 g/d (*p* = 0.005; 95%CI: −91 to −16 g/d).

### 3.3. Fasting Biochemical Measures and Mixed Meal Tolerance Test Responses

From fasting blood samples obtained throughout the intervention (Table 5, Table S1), HbA1c was not significantly lower after the 4-week TRE intervention (−0.2 ± 0.4%; *p* = 0.053). No differences were measured in fasting glucose, insulin, or lipid concentrations between Habitual and TRE periods. 

**Figure 4:**
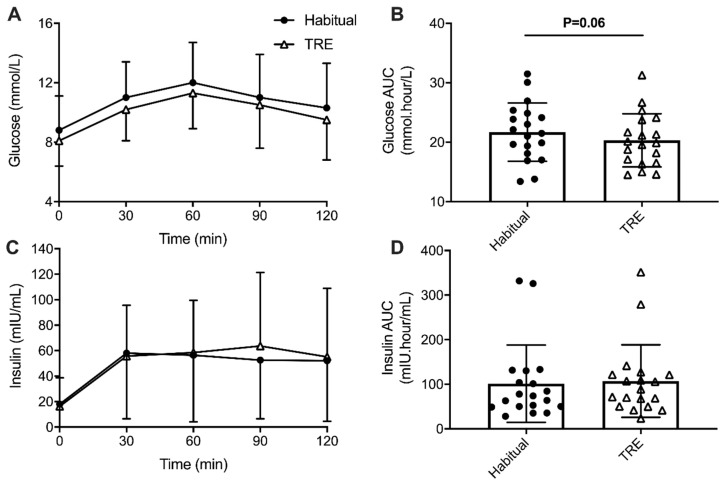
Concentrations and total area under the curve (AUC) of glucose (**A**,**B**) and insulin (**C**,**D**) in response to a mixed meal tolerance test (20% total daily energy requirements; 50% CHO, 30% fat, 20% protein) conducted at baseline (Habitual) and after a 4-week time-restricted eating (TRE) intervention for individuals with type 2 diabetes and overweight/obesity.

Blood samples were unable to be obtained throughout the MMTT for one participant, and thus data for this measure is from *n* = 18 participants. Mean glucose concentrations over the MMTT were significantly lower between Habitual and TRE (main effect of condition: *p* < 0.001) but glucose concentrations did not change differently over time between Habitual and TRE (Interaction effect; *p* = 0.97; Figure 4A). Fasting glucose levels at the beginning of the MMTT was not significantly lower after the TRE intervention (Habitual: 8.8 ± 2.2 mmol/L; TRE: 8.2 ± 1.7 mmol/L; −0.6 mmol/L, 95%CI: 0.1 to −1.3 mmol/L, *p* = 0.075), as did peak glucose during the MMTT (Habitual: 12.0 ± 2.8 mmol/L; TRE: 11.4 ± 2.4 mmol/L; −0.7 mmol/L, 95%CI: 0.1 to −1.4 mmol/L, *p* = 0.075). Total AUC for glucose was not significantly lower after the TRE intervention (*p* = 0.056; Figure 4B). There were no differences between Habitual and TRE periods in response to the MMTT for insulin concentrations (main effect of condition: *p* = 0.38) or total AUC insulin (*p* = 0.20; Figure 4C,D, respectively). Consequently, neither fasting nor peak insulin concentrations from the MMTT were not different between Habitual and TRE periods (*p* = 0.45 and *p* = 0.37, respectively).

### 3.4. Physiological Data

There were no differences between body mass, composition resting energy expenditure, or blood pressure measures between the Habitual period and the end of the TRE intervention period (Table 6). 

### 3.5. Psychological Data

#### 3.5.1. Psychological Wellbeing

Within-group comparisons indicated no clinically (effect sizes were small) or statistically significant changes on the DASS, the AQoL-8D or the PSQI, the EDE-Q or the CIA (Table 7).

#### 3.5.2. Cognitive Functioning

Due to high error rates compromising the validity of the test and test timing out, five participant data points were missing from the CBB. Due to the small sample size, it was determined that pairwise deletion was the most appropriate method for managing Cogstate missing data [46]. Within-group comparisons indicated significant improvement on the Groton Maze Learning Task post-intervention, with a large effect size, and significant decreased performance on the detection task, with a large effect size. There were no clinically (effect sizes were small) or statistically significant changes on the remaining subtests.

### 3.6. Qualitative Questionnaire Responses

Sixteen of the 19 participants completed post-intervention qualitative interviews (~14 ± 3 min). Thematic analysis revealed four themes related to participant experiences over the 4-week TRE intervention period: (1) difficulties with the morning or evening, (2) impact on eating behaviours and food choices, (3) emotional reactions, and 4) monitoring and accountability. A summary of the themes and sample quotes identified from participant interviews are outlined in Table 8.

## 4. Discussion

This is the first study to investigate the feasibility of TRE in individuals with T2D, and explore the implications of TRE on dietary intake, glycaemic control, physiological outcomes, and psychological wellbeing. We report that TRE was feasible on 5 days/week, with no adverse effects. We show that TRE was an attainable dietary strategy for individuals with T2D, reducing the daily eating window and decreasing dietary energy intake on adherent TRE days. These results provide a foundation for future research to explore long-term biopsychosocial impacts of TRE in individuals with T2D.

Based on objective time-stamped photo data, participants complied with the 10:00–19:00 h daily eating window for ~5 days/week (77%) during the intervention period, consistent with previous research assessing adherence based on self-report or food records [1,2]. Adults of similar age and weight to our cohort, but without diabetes, self-reported adhering to 8-h TRE for 8 weeks on 5.6 days/week [2]. In a 10-week study, middle-aged adults adhered to TRE (intake shifted 1.5 h later in morning and 1.5 h earlier in evening) on 2.5/4 days based on a single four-day food record [1]. Across our cohort, rate of adherence varied widely over the 4-weeks of TRE from 4% to 100% demonstrating that adherence to the time restriction was not always possible. Total energy intake, macronutrient distribution and the number of eating occasions did not change over the four-week TRE period. Further, participants reported being adherent on 43% of days deemed non-adherent by recorded timing, suggesting previous studies using self-reporting methodology may have overestimated adherence [2,5].

A reduction in reported energy intake was expected due to previous reports suggesting there may be up to 20% energy reduction in longer-term (10–16 week) TRE interventions [1,2,3,4]. However, the consistency in energy intake between the habitual and intervention periods in the current study suggests participants did not make substantial or additional dietary changes other than reducing the time window of eating and is reflected in the lack of change to physiological measures such as body mass. It is possible that this 4-week intervention was too brief to impact physiological measures, although a previous TRE intervention in older (>65 y) adults resulted in reduced body mass (−2.6 kg) after 4-weeks [47].

TRE reduced total reported energy intake by ~1000 kJ/d (~11%) on days when all eating occasions occurred within the prescribed eating window, due to decreased carbohydrate and alcohol consumption. Participants’ overall adherence during TRE was associated with a lower carbohydrate intake, results in line with previous research [3]. This suggests that TRE can reduce consumption of discretionary “time of day foods” (e.g., ice-cream, chocolate, and alcohol in the evenings), and reduce total energy intake. Consistent with self-reported lack of adherence due to social occasions in previous research [1], non-adherence occurred mostly on weekends. 

Participants consumed their total energy intake over ~11 h in the Habitual period compared to ~8.5 h in the TRE period. Energy consumption after 19:00 h contributed to the majority of the non-adherent occasions (63%), corresponding to the largest energy intake period over a day [3,33]. In men with overweight/obesity, consuming an earlier dinner (17:00 vs. 21:00 h) induced greater peak insulin concentration and a trend for lower glucose incremental area under the curve in previous research [44]. As there is evidence that eating even earlier (by 15:00 h) can improve *ß*-cell function and 24-h glucose concentrations [7,8], TRE may be a useful strategy to limit end of day eating and post-dinner snacking of discretionary foods (e.g., ice cream and chocolate) and alcohol. A head-to-head comparison of TRE schedules (i.e., late vs. early) in men at risk of prediabetes showed improved glucose tolerance in both TRE conditions after one week but the earlier (08:00–17:00 h vs. 12:00–20:00 h) window improved fasting glucose concentrations [9]. There is need to identify the most appropriate and achievable time window of energy intake to encourage healthy dietary practices and improved metabolic health.

The recruitment rate for this study, as a measure of feasibility, was low. Recruitment through an external company and the researcher-driven methods achieved similar rates of success, although a large proportion may have self-screened due to the perceived inability to adhere to the 10:00 to 19:00 h eating window, which could be overcome in future research by providing a guide of number of hours [1,3] rather than a specific TRE time period. The overall low rate of recruitment is likely to be directly related to the strict inclusion/exclusion criteria in order to have as homogenous participant cohort as possible to investigate physiological outcomes. As such, the results of the current study apply directly to adults with T2D, specifically those who are not on more than two oral hypoglycaemic agents or insulin or with uncontrolled hyperglycaemia (HbA1c > 9.5%).

While various measures of glycaemic control have been improved in previous *ad libitum* and energy-matched TRE interventions of varying durations (4 days to 12 weeks), all previous investigations have been in those with normal or impaired glucose tolerance [4,5,7,8,9,27,44,48]. Individuals with T2D have altered circadian patterns of glucose regulation [17,49], with greatest impairments to glucose tolerance in the morning compared to the evening in those with normal or impaired glucose tolerance. Furthermore, individuals with T2D are heterogenous in the response to glucose loads and, in the current investigation, participants varied in their baseline glycaemic control (HbA1c) and length since T2D diagnosis. Overall, there were small but non-significant improvements (reductions) in HbA1c concurrent with a reduction in total AUC glucose in response to a mixed meal tolerance test. However, the magnitude of change in HbA1c was smaller than that which would be deemed clinically significant (i.e., −0.5% HbA1c) and the duration of the intervention was shorter (1 mo) than the duration required (3 mo) see a measurable change in HbA1c. Importantly, the TRE intervention did not worsen glycaemic control despite energy intake remaining the same within a reduced time window of eating. Whether TRE per se as a dietary strategy can improve glycaemic control over that of typical dietary modification (i.e., reduction of energy intake and improvement of dietary quality) requires further investigation and over a longer duration. Further consideration of changes to physical activity either alongside or in comparison with TRE is also worthy of investigation [50]. However, as demonstrated by the adherent TRE days, TRE may induce some of the dietary changes that would be implemented in dietetic practices, such as reduction in carbohydrate and alcohol intake, which would lend to a hypothesis of further glycaemic improvements over a longer period of implementation.

This is the first study to assess the psychological and cognitive impacts of TRE in individuals with T2D. There were no clinically or statistically significant changes in psychological well-being, disordered eating cognitions or impairment, sleep quality, or quality of life. The lack of change in sleep quality variables may suggest that TRE did not impact sleep, supported by a similar study in those with overweight/obesity [51], although many of the participants in the current study reported appropriate sleep quantity and quality at baseline. Importantly, the lack of change in psychological well-being measures suggest that this TRE intervention did not negatively impact these outcomes. Psychological improvements observed in other dietary intervention studies [52,53,54,55] were not evident in the present study.

Cognitive functioning was assessed across five domains (processing speed, attention, visual learning, working memory, and executive functioning) with improvement on executive function and decreased performance on processing speed. Improvements in executive function are consistent with previous research showing improvements in this domain after fasting [56]. The deterioration of processing speed was unexpected as previous research suggests that processing speed is not affected by acute periods of fasting, although findings from the current study are reflective of the inconsistencies noted in reviews on the impact of fasting on cognitive functioning domains [57]. The lack of psychological improvement and inconsistent impacts on cognitive function may be due to the brevity of the intervention. Given the negative psychological and cognitive impacts of T2D and its management, the impact of psychological and cognitive impairment on successful self-management, and the potential for lifestyle interventions to improve psychological wellbeing and cognitive function, it is essential that future TRE studies comprehensively measure psychological wellbeing and cognitive function.

Four key themes arose from the qualitative interviews. First, participants reported difficulties managing either the morning or the evening. Specifically, “hunger” and “missing morning coffee” and/or eating before 19:00 h were difficult to integrate with family, social, or work commitments. Second, participants reported impacts of TRE on hunger, eating behaviours, and food choices with some reporting feeling hunger or concerns of feeling hungry, while others noted that they were not as hungry as expected. Some indicated that TRE assisted them to stop night-time eating, while others reported difficulties in avoiding night-time snacking due to negative emotions (e.g., stress or boredom). Some participants stated that TRE facilitated planning healthier, more sustainable meals, while others noted that because they had not planned, they ate convenient/"junk" food [1]. Third, participants reported both positive and negative emotional reactions to TRE, with some embracing the structure and routine while others felt stressed/anxious trying to comply. Finally, participants reported that self-monitoring increased their self-awareness and accountability of food intake. Collectively, our data suggest adherence and maintenance to TRE protocols may be improved if supported by simple health behaviour change strategies to counter some of the perceived and real barriers to TRE. 

There are several limitations of the current study. First, this was a non-randomised intervention and no control group was included. Second, our study was of short duration (4 weeks) which has limited the physiological changes that can be induced. The variability of the cohort due to strict eligibility criteria regarding medication and baseline eating time window may limit the generalisability of the results to a wider cohort of individuals with T2D. Further, the group recruited, as with any study of this nature, may have been a highly motivated group which does not represent the entire population of individuals with T2D. Adherence to the 9-h TRE may have been affected by strict restriction of caffeine only within the eating window (due to the known metabolic effects of caffeine [58]), requirements to log all dietary intake using photos and written or app food diaries (where dietary recordings are known to potentially alter dietary intake [59]), and purposefully only asking participants to restrict their eating window “on as many days each week as possible” (acknowledging that participants may be more likely to adhere if they feel in control [60]). Participants were specifically asked to follow the eating window restrictions on as many days as possible to allow for ownership of the dietary regime [60]. However, the robust dietary methodology/analysis and psychological assessments are strengths, which provide both qualitative and quantitative data to assist in improving future implementation of TRE practices. Of note, we would not expect such robust dietary data to be collected in longer interventions, where shorter (3–5 day) periods of records and photos collected intermittently used throughout longer interventions would be recommended. Further, the qualitative questionnaires provide novel insights as to how TRE is perceived by individuals with T2D.

## 5. Conclusions

In conclusion, 4-weeks of 9-h TRE is feasible and achievable for individuals with T2D, with the extent of adherence to TRE strongly influencing daily energy intake. Given the link between dietary intake, effective diabetes management and psychological/cognitive factors, it is critical future studies examining the efficacy of TRE as a dietary strategy for diabetes management continue to examine psychological and cognitive outcomes. Future interventions should assess the potential for TRE to facilitate dietary changes and achieve long-term adherence for individuals with T2D or at risk of developing T2D. 

## Figures and Tables

**Table 1:** Participant characteristics of participants with type 2 diabetes who completed the study.

	All (*n* = 19)	Males (*n* = 9)	Females (*n* = 10)
Age (y)	50.2 ± 8.9	48.7 ± 10.0	51.6 ± 8.0
Body mass (kg) *	99.7 ± 12.7	99.8 ± 12.6	101.1 ± 12.5
Height (m)	1.71 ± 0.09	1.78 ± 0.04	1.66 ± 0.08
BMI (kg/m^2^)	34.4 ± 4.8	31.4 ± 3.4	36.8 ± 4.8
Baseline HbA1c (% (mmol/mol))	7.6 ± 1.1 (59 ± 12)	7.8 ± 1.1 (66 ± 12)	7.1 ± 0.7 (54 ± 8)
Years diagnosed T2D	3.4 ± 3.1	4.7 ± 3.5	2.6 ± 2.5
MEQ-SA	56 ± 11	55 ± 11	57 ± 11

Data are mean ± SD. BMI: body mass index; HbA1c: glycated haemoglobin; MEQ-SA: morning-eveningness questionnaire, where a score of 70–86 indicates “definite morning” types (*n* = 2), 59–69 indicates “moderate morning” types (*n* = 5) or 42–58 for “intermediate” types (*n* = 10), or 31–41 for “moderate evening” types (*n* = 2); T2D: type 2 diabetes. * measured whilst fasted, on scales at body composition scan (light clothing/gowned).

**Table 2:** Overview of dietary intake for Habitual (13.8 ± 0.4 days) vs. time-restricted eating (TRE; 27.6 ± 1.4 days) periods as assessed from daily dietary food records for individuals with type 2 diabetes and overweight/obesity.

	Habitual Period	TRE Period	*p*
Energy (kJ/d)	8399 ± 2864	8566 ± 2704	0.42
Protein (g/d)	95 ± 35	97 ± 37	0.66
Protein (% of TEI)	19.6 ± 5.7	19.2 ± 5.6	0.29
Total fat (g/d)	94 ± 45	96 ± 42	0.67
Saturated fat (g/d)	34 ± 18	35 ± 18	0.53
Polyunsaturated fat (g/d)	16 ± 10	16 ± 10	0.65
Monounsaturated fat (g/d)	36 ± 20	37 ± 18	0.78
Total fat (% of TEI)	41.4 ± 10.5	41.8 ± 11.2	0.68
Carbohydrate (g/d)	175 ± 86	181 ± 86	0.37
Carbohydrate (% of TEI)	34.9 ± 11.6	35.3 ± 12.1	0.65
Sugars (g/d)	55 ± 31	55 ± 33	0.99
Fibre (g/d)	25 ± 11	25 ± 12	0.86
Alcohol (g/d *)	4 ± 14	4 ± 15	0.89
Alcohol (% of TEI)	1.4 ± 4.8	1.3 ± 4.4	0.76
Vitamin C (mg/d)	70 ± 54	78 ± 70	0.13
Sodium (mg/d)	2834 ± 1475	2762 ± 1352	0.49
Potassium (mg/d)	2769 ± 1009	2738 ± 981	0.68
Calcium (mg/d)	830 ± 449	822 ± 505	0.84

Data are mean ± SD. TEI: total energy intake, * Includes days of non-alcohol consumption.

**Table 3:** Comparison of self-reported (written, handbook) versus time-stamped photo adherence to the 10:00–19:00 h TRE period (±15 min i.e., 09:45–19:15 h).

*n*, Days	Adherent (Time Data)	Non-Adherent (Time Data)	Total Self-Report
Adherent (self-report)	302	55	357 (68%)
Non-adherent (self-report)	23	73	96 (18%)
Not answered	53	18	71 (14%)
Total time data	378 (72%)	146 (28%)	524 (100%)

**Table 4:** Comparison of dietary intake for adherent and non-adherent days during the time-restricted eating (TRE) period as assessed from daily dietary food records for individuals with type 2 diabetes and overweight/obesity.

	TRE-Adherent Days (*n* = 378)	TRE-Non Adherent Days (*n* = 146)	*p*
Energy (kJ/d)	8278 ± 2556	9312 ± 2936	<0.001
Protein (g/d)	97 ± 36	96 ± 38	0.75
Protein (% of TEI)	20 ± 6	18 ± 6	<0.001
Total fat (g/d)	96 ± 43	96 ± 39	0.99
Saturated fat (g/d)	35 ± 18	35 ± 17	0.97
Polyunsaturated fat (g/d)	16 ± 9	16 ± 10	0.72
Monounsaturated fat (g/d)	37 ± 18	36 ± 17	0.79
Total fat (% of TEI)	43 ± 11	39 ± 10	<0.001
Carbohydrate (g/d)	167 ± 78	216 ± 96	<0.001
Carbohydrate (% of TEI)	34 ± 12	39 ± 11	<0.001
Sugars (g/d)	52 ± 33	62 ± 32	0.001
Fibre (g/d)	24 ± 11	27 ± 14	0.006
Alcohol (g/d *)	2 ± 7	9 ± 24	<0.001
Alcohol (% of TEI)	1 ± 3	3 ± 7	<0.001
Vitamin C (mg/d)	77 ± 66	81 ± 80	0.51
Sodium (mg/d)	2705 ± 1297	2908 ± 1479	0.12
Potassium (mg/d)	2682 ± 922	2883 ± 1111	0.04
Calcium (mg/d)	812 ± 503	850 ± 513	0.44

Data are mean ± SD. * Includes days of non-alcohol consumption; days when all eating occasions occurred between 09:45 and 19:15 h were considered adherent.

**Table 5:** Concentrations of blood metabolites measured after a >10 h fast in individuals with type 2 diabetes and overweight/obesity measured at the end of the 2-week Habitual period and after the 4-week TRE intervention period.

	Habitual Period	TRE Period	*d*	*p* *
HbA1c (%) (mmol/mol)	7.6 ± 1.1 (60 ± 12)	7.4 ± 1.0 (58 ± 11)	0.17	0.053
Glucose (mmol/L)	8.4 ± 2.3	8.1 ± 1.8	0.18	0.29
Insulin (mIU/mL)	15.0 ± 15.2	17.7 ± 25.2	0.13	0.09
Total cholesterol (mmol/L)	4.6 ± 0.9	4.5 ± 0.8	0.13	0.16
HDLC (mmol/L)	1.1 ± 0.3	1.1 ± 0.3	0.01	0.75
LDLC (mmol/L)	2.6 ± 0.9	2.5 ± 0.8	0.17	0.22
Triglycerides (mmol/L)	1.8 ± 0.7	1.8 ± 0.8	0.00	0.78

Data are mean ± SD, *d* = effect size. Key: HbA1c, glycated haemoglobin; HDLC, high-density lipoprotein cholesterol; LDLC, low-density lipoprotein cholesterol; TRE, time-restricted eating. * Statistical analysis: linear mixed model testing effects of time with all blood sampling timepoints included (full data available in Table S1), with effect sizes (cohens *d*).

**Table 6:** Overview of physiological variables of individuals with type 2 diabetes and overweight/obesity measured at the end of the 2-week Habitual period and after the 4-week TRE intervention period.

	Habitual Period	TRE Period	*d*	*p*
Total body mass (kg) *	98.9 ± 12.7	98.1 ± 12.8	0.07	0.84
Lean mass (kg) *	56.3 ± 9.3	56.2 ± 9.6	0.01	0.97
Fat mass (kg) *	39.8 ± 10.3	39.0 ± 10.3	0.07	0.84
Bone mass (kg) *	2.8 ± 0.4	2.8 ± 0.3	0.00	1.00
Resting energy expenditure (kcal/d) *	1889 ± 298	1889 ± 310	0.00	1.00
Blood pressure (BP)				
Systolic BP (mmHg)	131 ± 12	126 ± 7	0.51	0.12
Diastolic BP (mmHg)	84 ± 6	80 ± 4	0.54	0.11
Heart rate (beats per min)	72 ± 15	66 ± 10	0.52	0.12

Data are mean ± SD, *d* = effect size. TRE, time-restricted eating. * *n* = 18 for each measure as one participant could not undergo their post-TRE DXA due to equipment being unavailable and one participant could not undergo any RMR testing due to claustrophobia.

**Table 7:** Mean differences between outcome scores of the DASS, AQoL, PSQI, CBB, EDE-Q and CIA at Habitual and post-TRE intervention time points.

Domain	Habitual	Post-TRE Intervention		t	*d*	*p*
Depression, Anxiety and Stress Scale (DASS)						
Depression	6.42 ± 8.90	7.05 ± 9.71		−0.277	0.07	0.79
Anxiety	4.53 ± 4.56	6.42 ± 7.32		−1.158	0.32	0.28
Stress	7.68 ± 7.24	8.31 ± 8.51		−0.287	0.08	0.78
Assessment of Quality of Life (AQoL-8D)						
Independent living	0.90 ± 0.12	0.92 ± 0.13		−0.919	0.16	0.37
Happiness	0.77 ± 0.15	0.76 ± 0.15		0.014	0.07	0.99
Mental health	0.64 ± 0.13	0.63 ± 0.14		0.253	0.00	0.80
Coping	0.77 ± 0.14	0.77 ± 0.17		−0.01	0.00	0.92
Relationship value	0.75 ± 0.18	0.75 ± 0.21		−0.08	0.00	0.94
Self-worth	0.83 ± 0.19	0.79 ± 0.17		1.15	0.22	0.27
Pain value	0.74 ± 0.26	0.74 ± 0.26		−0.10	0.00	0.92
Senses	0.87 ± 0.08	0.87 ± 0.10		−0.14	0.00	0.89
Pittsburgh Sleep Quality Index (PSQI)						
Quality	1.26 ± 0.87	1.21 ± 0.63		0.252	0.07	0.80
Latency	1.37 ± 1.26	1.21 ± 1.13		0.718	0.13	0.48
Duration	0.84 ± 0.76	0.84 ± 0.69		0.000	0.00	1.00
Efficiency	0.74 ± 0.99	0.68 ± 1.00		0.170	0.04	0.87
Disturbance	1.42 ± 0.51	1.53 ± 0.70		−0.697	0.18	0.49
Medication	0.42 ± 0.61	0.31 ± 0.47		0.567	0.20	0.58
Daytime sleepiness	0.95 ± 0.85	0.89 ± 0.93		0.224	0.07	0.83
Global	7.00 ± 4.29	6.68 ± 3.84		0.275	0.08	0.79
Cogstate Brief Battery (CBB)						
Groton Maze Learning	62.42 ± 20.14	50.42 ± 14.81		3.334	0.69	0.004
Identification ª	2.75 ± 0.11	2.73 ± 0.05		0.539	0.25	0.60
Detection ᵇ	2.52 ± 0.07	2.57 ± 0.07		−2.616	0.71	0.02
One Card Learning	0.91 ± 0.16	0.93 ± 0.14		−0.534	0.13	0.60
Two Back ᶜ	1.07 ± 0.35	1.11 ± 0.34		−0.523	0.12	0.61
Eating Disorders Examination Questionnaire (EDE-Q)						
Restricted	1.97 ± 1.35	1.84 ± 1.44		0.429	0.09	0.67
Eating	1.24 ± 1.91	1.62 ± 1.98		−1.421	0.20	0.17
Shape	2.42 ± 1.52	2.33 ± 1.53		0.268	0.06	0.78
Weight	2.20 ± 1.21	2.27 ± 1.45		−0.352	0.05	0.73
Global	1.96 ± 1.28	2.02 ± 1.36		−0.345	0.05	0.72
Clinical Impairment Assessment (CIA)						
Total	6.31 ± 10.23	7.89 ± 12.16		−1.119	0.14	0.25

Key: t = z-statistic; *d* = effect size; ª *n* = 17; ᵇ *n* = 16; ᶜ *n* = 18.

**Table 8:** Qualitative themes from in-person interviews (*n* = 16) conducted post-TRE intervention.

Theme	Sub-Theme	Sample Quote
Mornings or evenings were difficult (of note, participants generally reported having trouble with the morning OR the evening, not both)	Mornings: starting eating/drinking later was difficult because of hunger or missing the morning coffee and feeling like it impacted on the ability to function.	*“Morning things... if you have morning things, then the 10 am breakfast is a bit difficult to do.”* *“Coffee was a big factor... I couldn’t function too well in the morning at all.”*
	Evenings: finishing eating earlier was difficult because of family, social (particularly on the weekends) or work commitments, or hunger later in the evening.	*“Whether I could go for dinner or saying no to doing things especially during the week... because I would have to do them really early.”* *“I wasn’t hungry at night... I did have a couple of nights where I did feel hungry, but it started to dissipate, and I felt less hungry when I woke up.”*
Impact on hunger, eating behaviour and food choices	Hunger: Feeling hungry, or concerned about being hungry, or, feeling less hungry than expected particularly over time.	*“You think 10 a.m. is a long time and then sometimes you didn’t have enough the night before and didn’t eat a lot and the next day you’re like, ’I should’ve eaten more last night... I am hungry.’”* *“Well at first, I thought I was going to struggle with being hungry, then coming to the conclusion of it, yeah, I was a lot more satisfied and not as hungry and didn’t feel like I needed to eat all the time.”*
	Emotional eating and night-time snacking: response to not being able to eat to manage negative emotions (e.g., stress, boredom), and breaking the habit of night-time snacking.	*“Probably a little better for it [TRE]. I am prone to eating because I don’t know if boredom or something but when it’s there, you go and snack.”* *“I guess the main thing for me was I could avoid late night snacking... I was always struggling to stop that, but I guess it [TRE] helped me a lot to stop it totally”*
	Healthy food or convenient food: either planning ahead to eat healthier, more substantiating meals to avoid evening hunger, or not having healthy options organised and therefore eating convenient food in order to eat before 1900 h.	*“…by the time I got home, it was very difficult to cook dinner at that time. So, unless I had something pre-prepared, I was going to eat junk. I would have rather eaten slightly later than eat junk.”* *“I did sort of think I probably need to eat something healthier that is going to keep me going for a little bit longer because I am not going to have another opportunity to have anything else.”*
Positive or negative emotional reactions	Positive reaction: felt good about the routine, appreciated the structure, felt in control, felt had more time, did not feel like a diet (because asked to change when eating, but not what is eaten).	*“I found it suited my lifestyle... I tend to be an organised person.”* *“Enjoyed preparing my meals early and finish up early for the day. That was the best thing.”* *“Other diets you need to make a choice of what you need to eat and counting everything you want to eat, but this one I just eat what I want to eat and how much. I didn’t have to do all the counting.”*
	Negative reaction: found it stressful, felt anxious about sticking to the routine.	*“There was always kind of a stress I have to do it in between this time.”*
Monitoring and Accountability	Self-monitoring: increased self-awareness and accountability, identifying patterns and relationships (e.g., eating and blood glucose).	*“I think some of that accountability of thinking about what you’re eating and having to report it all.”* *“The data for me is a big thing... seeing how my blood sugars will react to the foods.”*

## Supplementary Materials

The following are available online at https://www.mdpi.com/2072-6643/12/11/3228/s1, Qualitative Interview Questions; Figure S1: Box plots depicting participant (A–S) and cohort (All; *n* = 19) dietary intakes of (a) energy, (b) protein, (c) total fat, (d) saturated fat, (e) carbohydrate, (f) sugars, (g) fibre, (h) alcohol, (i) sodium, (j) potassium, (k) vitamin C, and (l) calcium throughout the Habitual (2 weeks; white bars and circles) and TRE (4 weeks; grey bars and black triangles) intervention periods; Figure S2: Dietary distribution and macronutrient distribution as a proportion of energy intake in hourly blocks throughout the Habitual (2 week) and TRE (4 week) periods; Table S1: Concentrations of blood metabolites measured after a >10 h fast in individuals with type 2 diabetes and overweight/obesity measured at the end of the 2-week Habitual period and weekly during the 4-week TRE intervention period.
