# Supplementary material for: Time-Restricted Eating as a Nutrition Strategy for Individuals with Type 2 Diabetes: A Feasibility Study
Source: Nutrients. 2020 Oct 22;12(11):3228. doi: 10.3390/nu12113228 (PMC7690416; doi:10.3390/nu12113228)
Supplement: Supplementary file 1 [file nutrients-12-03228-s001.pdf]

Supplementary Material

Materials and Methods

Qualitative Interview Questions

1. Based on your experience throughout the intervention period of consuming food between 10 am and 7 pm, what are your general thoughts and comments about such a dietary pattern?
2. What did you enjoy about the TRE intervention period?
3. What didn't you enjoy about the TRE intervention period?
4. Did you experience any barriers to adhering to a time-restricted eating pattern? If so, please comment.
5. What did you find appealing about the time-restricted eating pattern?
6. How did the changing the times of eating (i.e. the time-restricted eating pattern) make you feel?
7. Considering your home/work/life setting, on a scale of 1-10 (1 being not possible, and 10 being most definitely possible), how possible did you find it to adhere to a time-restricted eating approach to eating with the researcher support? And why did you give that rating?
8. Considering your home/work/life setting, on a scale of 1-10 (1 being not possible, and 10 being most definitely possible), how possible would you find it to adhere to a time-restricted feeding approach to eating without the researcher support (i.e. from here on)? And why did you give that rating?
9. How did you find the eating patterns influenced your hunger levels? Were there particular times of the day you were really hungry? Did this change across the four weeks of the TRE intervention?
10. How does a TRE pattern compare to other "diets" you have tried?
11. Do you think your food choices changed when you changed the time of eating? Was this a conscious or unconscious decision (i.e. did you mean to change your food choices, or did it naturally happen as a result of the time)?
12. How or in what way do you feel the TRE changed your overall health?
13. Any other comments, thoughts or opinions regarding the dietary approach of consuming food across a 9-hour window each day?

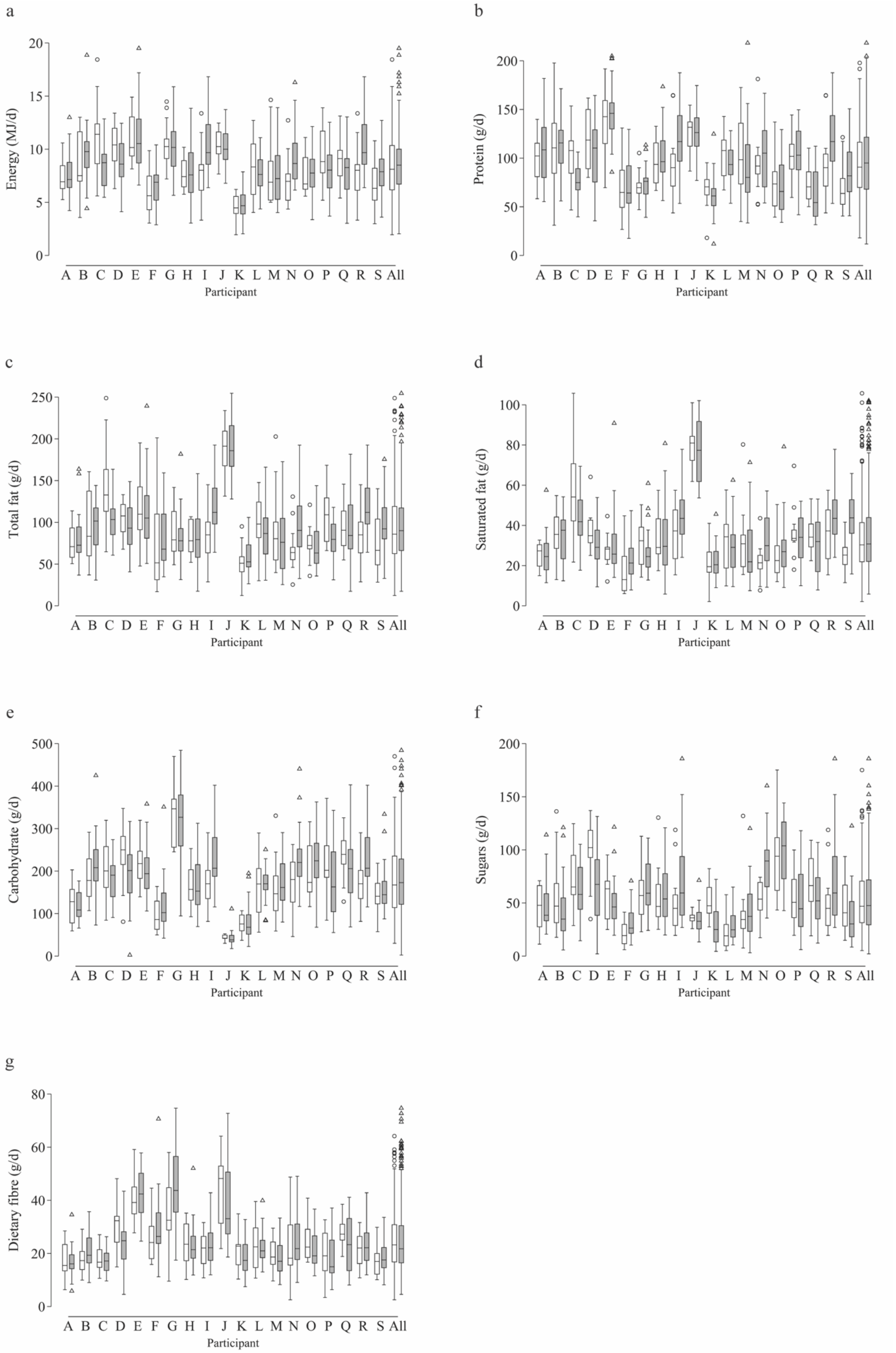
a
Energy (MJ/d)
0
5
10
15
20
A B C D E F G H I J K L M N O P Q R S All
Participant
b
Protein (g/d)
0
50
100
150
200
A B C D E F G H I J K L M N O P Q R S All
Participant
c
Total fat (g/d)
0
50
100
150
200
250
A B C D E F G H I J K L M N O P Q R S All
Participant
d
Saturated fat (g/d)
0
20
40
60
80
100
A B C D E F G H I J K L M N O P Q R S All
Participant
e
Carbohydrate (g/d)
0
100
200
300
400
500
A B C D E F G H I J K L M N O P Q R S All
Participant
f
Sugars (g/d)
0
50
100
150
200
A B C D E F G H I J K L M N O P Q R S All
Participant
g
Dietary fibre (g/d)
0
20
40
60
80
A B C D E F G H I J K L M N O P Q R S All
Participant

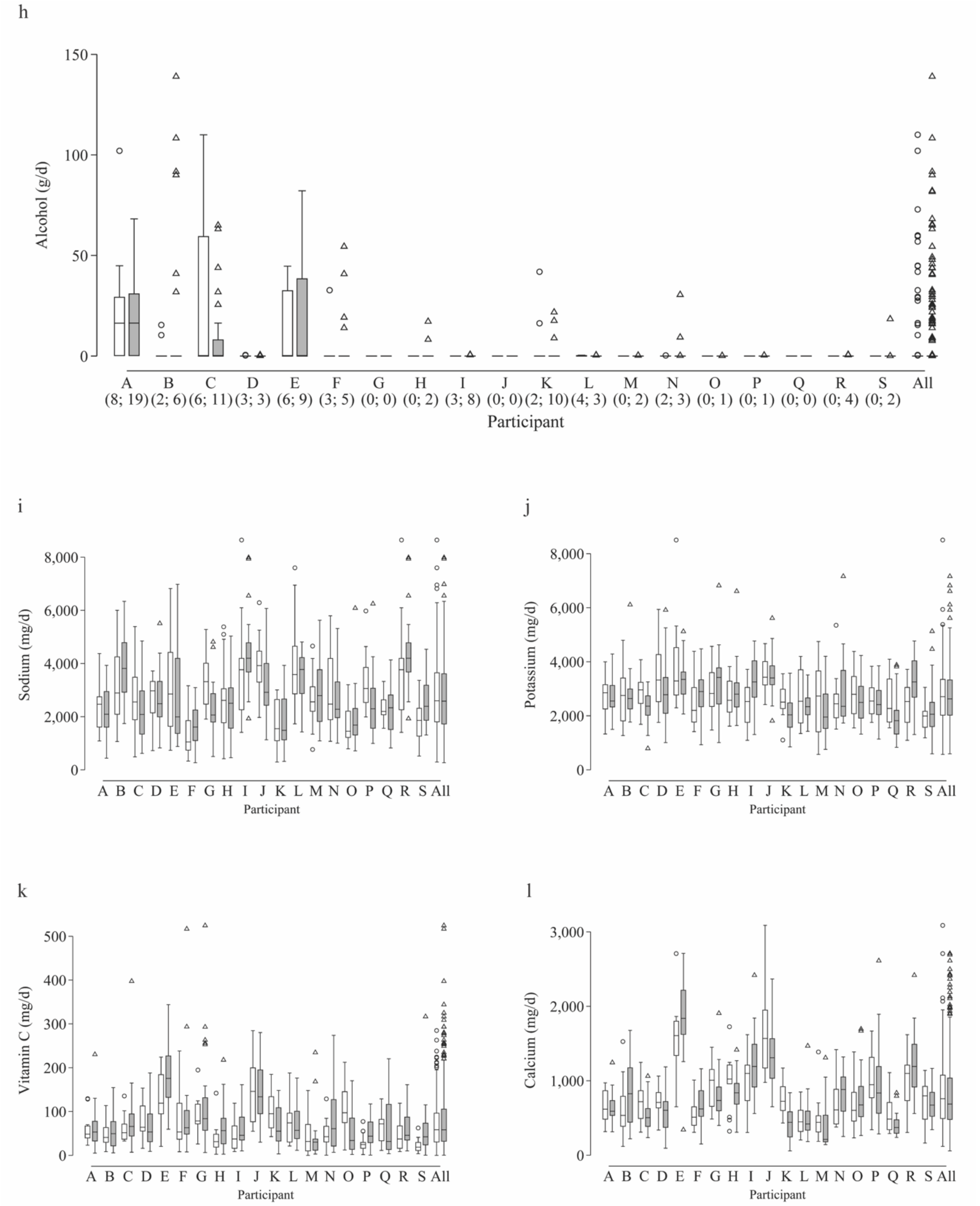

Figure S1. Box plots depicting participant (A-S) and cohort (All; n=19) dietary intakes of (a) energy, (b) protein, (c) total fat, (d) saturated fat, (e) carbohydrate, (f) sugars, (g) fibre, (h) alcohol, (i) sodium, (j) potassium, (k) vitamin C, and (l) calcium throughout the Habitual (2 wk; white bars and circles) and TRE (4 wk; grey bars and black triangles) intervention periods.

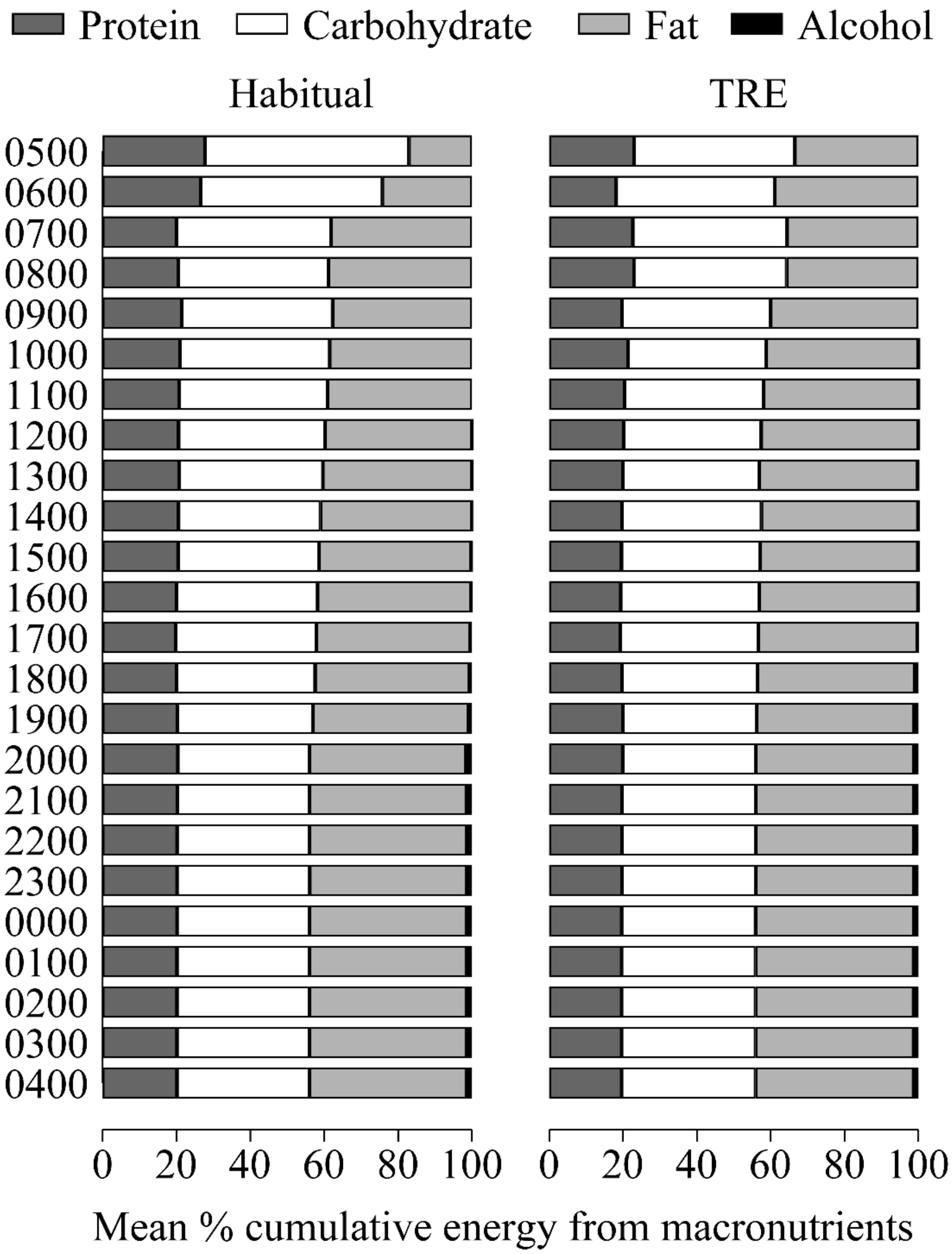

Figure S2. Dietary distribution and macronutrient distribution as a proportion of energy intake in hourly blocks throughout the Habitual (2 wk) and TRE (4 wk) periods.

Table S1. Concentrations of blood metabolites measured after a >10 h fast in individuals with type 2 diabetes and overweight/obesity measured at the end of the 2-wk Habitual period and weekly during the 4-wk TRE intervention period.

	**Habitual period**	**TRE period**				***P-value***
		**Week 1**	**Week 2**	**Week 3**	**Week 4**	
HbA1c (%) (mmol/mol))	7.6 ± 1.1 (60 ± 12)	--	--	--	7.4 ± 1.0 (58 ± 11)	0.053
Glucose (mmol/L)	8.4 ± 2.3	8.2 ± 2.4	8.1 ± 2.1	7.9 ± 1.6	8.1 ± 1.8	0.29
Insulin (mIU/mL)	15.0 ± 15.2	22.7 ± 36.5	15.9 ± 18.3	16.2 ± 19.0	17.7 ± 25.2	0.09
Total cholesterol (mmol/L)	4.6 ± 0.9	4.6 ± 0.8	4.5 ± 0.9	4.6 ± 0.9	4.5 ± 0.8	0.16
HDLC (mmol/L)	1.1 ± 0.3	1.1 ± 0.3	1.2 ± 0.3	1.2 ± 0.3	1.1 ± 0.3	0.75
LDLC (mmol/L)	2.7 ± 0.9	2.6 ± 0.9	2.6 ± 0.9	2.6 ± 0.9	2.5 ± 0.8	0.22
Triglycerides (mmol/L)	1.8 ± 0.7	1.7 ± 0.5	1.8 ± 0.8	1.8 ± 0.7	1.8 ± 0.8	0.78

Key: HbA1c, glycated haemoglobin; HDLC, high-density lipoprotein cholesterol; LDLC, low-density lipoprotein cholesterol. Statistical analysis methods used was a linear mixed model testing effects of time.
